# Advanced removal of Reactive Yellow 84 azo dye using functionalised amorphous calcium carbonates as adsorbent

**DOI:** 10.1038/s41598-022-07134-2

**Published:** 2022-02-24

**Authors:** Loredana Brinza, Andreea Elena Maftei, Sorin Tascu, Florin Brinza, Mariana Neamtu

**Affiliations:** 1 Department of Exact Sciences and Natural Sciences, Institute of Interdisciplinary Research, Alexandru Ioan Cuza University of Iasi, 11, Carol I Bvd Iasi, 700506 Iasi, Romania; 2 Research Center on Advanced Materials and Technologies, Department of Exact and Natural Science, Institute of Interdisciplinary Research, Alexandru Ioan Cuza University of Iasi, 700506 Iasi, Romania; 3 Faculty of Physics, Alexandru Ioan Cuza University of Iasi, 700506 Iasi, Romania

**Keywords:** Environmental chemistry, Environmental sciences

## Abstract

Two environmentally friendly organics (ethylenediaminetetraacetic acid, EDTA and its easier biodegradabe isomer, ethylenediamine-N, N′-disuccinic acid, EDDS) were used to dope calcium carbonate (CC) nanoparticles intending to increase their adsorptive properties and evaluate adsorption performance (uptake capacity and removal efficiency) for the persistent Reactive Yellow 84 azo dye. Easily synthesized nanomaterials were fully characterized (morphology and size, mineralogy, organic content, surface area, pore size and hydrodynamic diameter). RY84 removal was performed using two consecutive processes: photodegradation after adsorption. The CC-EDTA particles were most efficient for dye removal as compared to the plain and CC-EDDS particles. Adsorption kinetics and isotherms were considered for the CC-EDTA system. 99% removal occurred via adsorption on 1 g/L of adsorbent at 5 mg/L dye concentration and pH of 8 and it decreased to 48% at 60 mg/L. Maximum uptake capacity as described by Langmuir is 39.53 mg/g. As post-adsorption, under UVA irradiation, in the presence of 40 mmol/L H_2_O_2_, at dye concentration of 10 mg/L the highest degradation was 49.11%. Substantial decrease of adsorption (ca. 4 times) and photodegradation (ca. 5 times) efficiencies were observed in wastewater effluent as compared to distilled water. The results have important implications to wastewater treatments and appropriate decisions making for the choice of treatment process, process optimization and scaling up to pilot and industrial levels.

## Introduction

Nowadays, textile industry received significant attention owing to various type of dyes that often contains highly toxic metal complexes^1,2^. Dyes containing wastewaters are toxic and carcinogenic posing a threat to all living organisms on the trophic chain, including human health^3,4^. In addition, the dyes containing effluents from other industries such as food manufacturing, leather processing, paper industry, printing, paints and cosmetics pose also a critical concern to the environment by contributing to their discharge in fresh waters^5,6^. The most widely and hazardous dyes used in textile industry are azo dyes^7^. They are an aromatic complex compounds being resistant to wastewater handling methods due to their low degradability^8^. Reactive yellow 84 (RY84) is one of reactive textile dye largely used in textile finishing processes^9^. Therefore, their removal form wastewater has a major importance and need to be done before it can be discharged to surface waters.

Different treatment processes such as adsorption, membrane separation, physical and chemical coagulation, advanced oxidation processes (AOP), and biodegradation have been investigated to be applied in wastewater treatment^4,10–18^. The adsorption technology have proved to be the most suitable process in separation and removal of dyes from wastewater due to the economic feasibility and high performance^4,10^. An economic and environmentally feasible adsorbent should possess several important features such as: easily available, non-toxic, reusable, high stability, high selectivity for pollutants of interests, etc. Nanoparticles are promising a greater adsorption capacity due to the large specific surface area, sufficient active pores, inexpensive costs and non-toxicity^9^. Among economic minerals are calcium carbonate (CC) nanoparticles which so far were only tested for fluoride ions removal from aqueous solution^19^. As CC nanoparticles are easily available (one step and rapid synthesis) and green materials (their synthesis uses nontoxic raw materials), its uptake capacity for the removal of a specific azo dye worth to be examined. In addition, the CC nanoparticles have the advantage that they can be used on a pH interval (neutral to basic) that is close to the pH of the dye industrial effluent, thus it does not need major pH adjustment.

Nowadays, locking for new environmentally friendly and efficient materials for pollutants uptake, the trending is the use of various processes for particles functioning to improve the uptake capacity of pollutants uptake^1,5,9,20,21^. Accounting on that, this study considers the effect of two nontoxic organic compounds, such as ethylenediaminetetraacetic acid (EDTA) and ethylenediamine-N,N′-disuccinic acid (EDDS) on the CC nanoparticles surface properties, morphology and size, as potential nanoparticles functioning agents. Additionally, their ability to work as (1) scaffolding matrix for the CC structure and (2) potential role as stabilizers of the initial amorphous phase, called amorphous calcium carbonates (ACC), of the final adsorbent material, were also considered as current study hypothesis. The hypotheses that the ACC features a very high surface area as opposed to the ACC transformation end members^22–25^, and that the presence of organics in ACC synthesis may potentially preserve the ACC presence in the final products^26–29^ or will favour the formation of vaterite (higher surface area then calcite) beside calcite and will lead to an increase the surface properties of the final synthesis products, drive our interest in investigating calcium carbonates uptake capacity for an azo dye, named reactive yellow 84, RY84.

This particular dye poses a special interest due to its low degradability and low uptake on various sorbents which is manly given by its high molecular weight. Thus, the RY84 was chosen to be investigated in this study due to its refractoriness and minimal adsorption studies so far.

Unfortunately, at some industrial sites, depending on the pollutants load and type, some classic methods used could transfer the contaminant from wastewater to solid wastes. Therefore, in these cases other treatments are imposed to be applied for dye removal. Thus, advanced oxidation processes (AOP) are a second choice for dyes removal from wastewater via their total or partial degradation, ideally to invasive compounds such as CO_2_ and H_2_O^30–32^. AOP using UV irradiation and hydrogen peroxide (UV/H_2_O_2_) give good results in degradation of harmful contaminants and in wastewater treatment^17,33–38^. Therefore, this process could be considerate as a suitable post-treatment process in depollution of effluents from textile dyeing. Beside this, the method also has offered an environmental friendly perspective because it allows process water to be safety discharged with no other secondary products in surface waters or reused back in the industrial process^36,39^.

The textile and finishing industries use large amounts of water and recycling of wastewater could be an opportunity to decrease the cost of water management and could benefit environmental and natural resources. This study presents for the first time the synthesized calcium carbonate nanoparticles in the absence and in the presence of two organic components, applied as adsorbents for dye removal from aqueous solution. Further, studies of RY84 photodegradation post adsorption are carried out to improve its removal. Therefore, this study aims to combine subsequently adsorption and AOP processes (UV/H_2_O_2_ oxidation) for the removal of the most resistant and recalcitrant azo dye, RY84. The objectives of this research are to investigate the optimal conditions for dye removal via both processes.

## Results and discussion

### Nanoparticles synthesis and characterization

#### SEM microscopy

**Figure 1:**
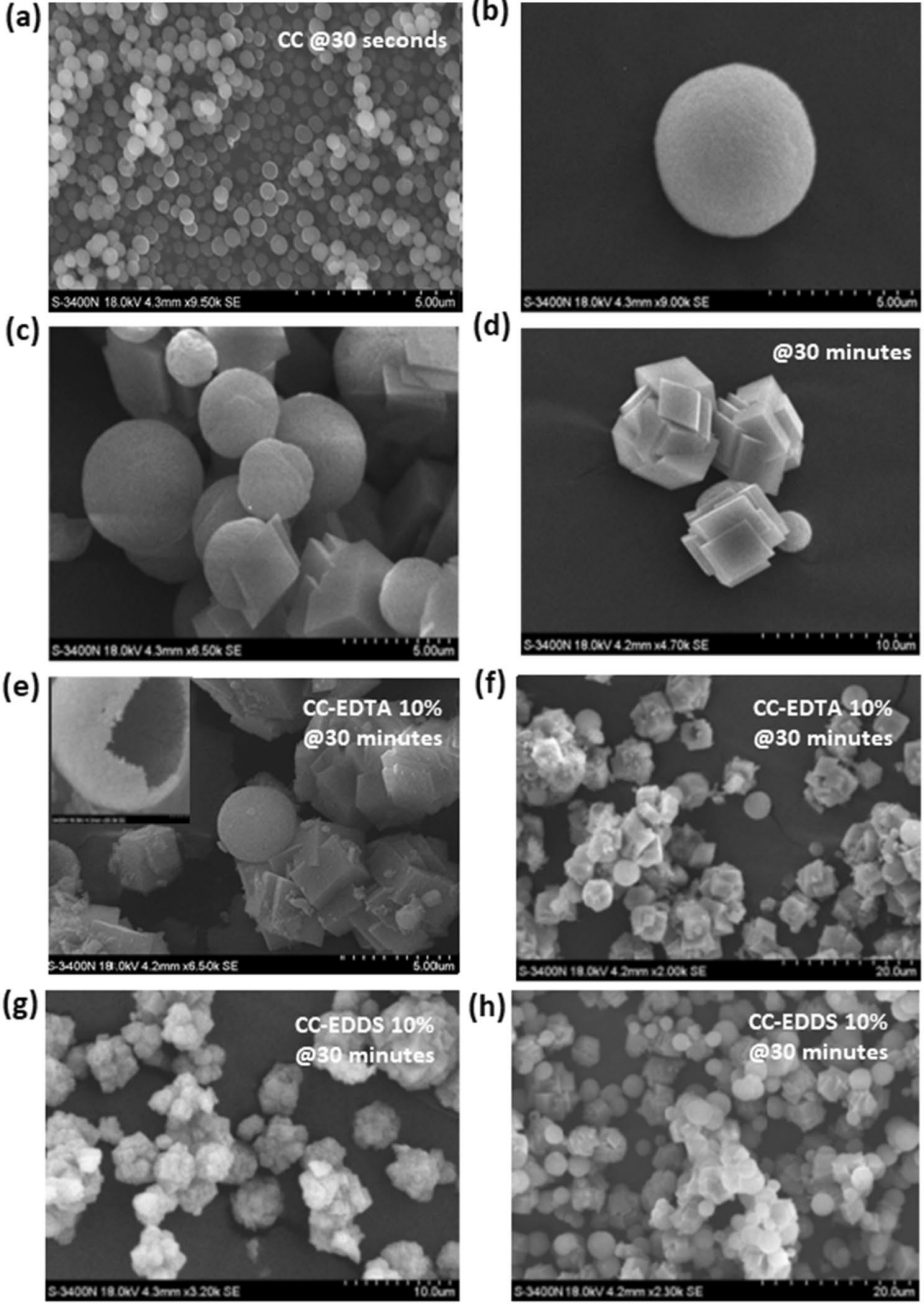
SEM images of carbonates particles synthesis in the absence and in the presence of EDTA 10% and EDDS 10%.

Figure 1 shows the scanning electron microscopic images (SEM) of synthesized carbonates in the presence and the absence of 10% EDTA and EDDS organics. Figure 1a, displays the spherical nanoparticles with average size of 50 nm. These nanoparticles are very unstable as they were present in solution only few seconds. The image was taken from a sample which was quenched at 30 s during the synthesis of carbonates without organics. Figure 1b displays a vaterite spheric particle of ca 5–8 microns in size, which was the second metastable polymorph intermediate formed in the system during synthesis. Interestingly, vaterite spheres have a rough surface which seems to suggest that they are made of ACC. This observation could be supported by another image captured for the EDTA system (see inset of Fig. 1e) which show a broken empty sphere of vaterite. Figure 1c and d display the morphology shape and size of the carbonates polymorphs that are formed via an amorphous calcium carbonate and vaterite as metastable intermediates. They are mainly calcite rhombohedral particles of ca 5 microns size with rather neat phases and minor traces of vaterite.

SEM micrographs of calcium carbonates synthetized in the presence of organics show that the addition of EDTA 10% (Fig. 1e and f) and EDDS 10% (Fig. 1g and h) led to a change in particles morphology and size. Thus, 10% organics added to their synthesis seems to have worked as a scaffolding making the calcite surface rougher (more panes and corner), which may have a positive impact on surface properties (i.e., surface area) as adsorbent.

The calcium carbonates possess different morphologies such as calcite, usually found as rhombohedral particles, and vaterite that has a hexagonal structure and here appeared to have a spherical structure and also find in other studies in the literature^22,24,40^. The features of the final products obtained in this study are well defined and consisted spherical shape of vaterite and rhombohedral phase of calcite.

#### XRD, Raman and FTIR spectroscopies

**Figure 2:**
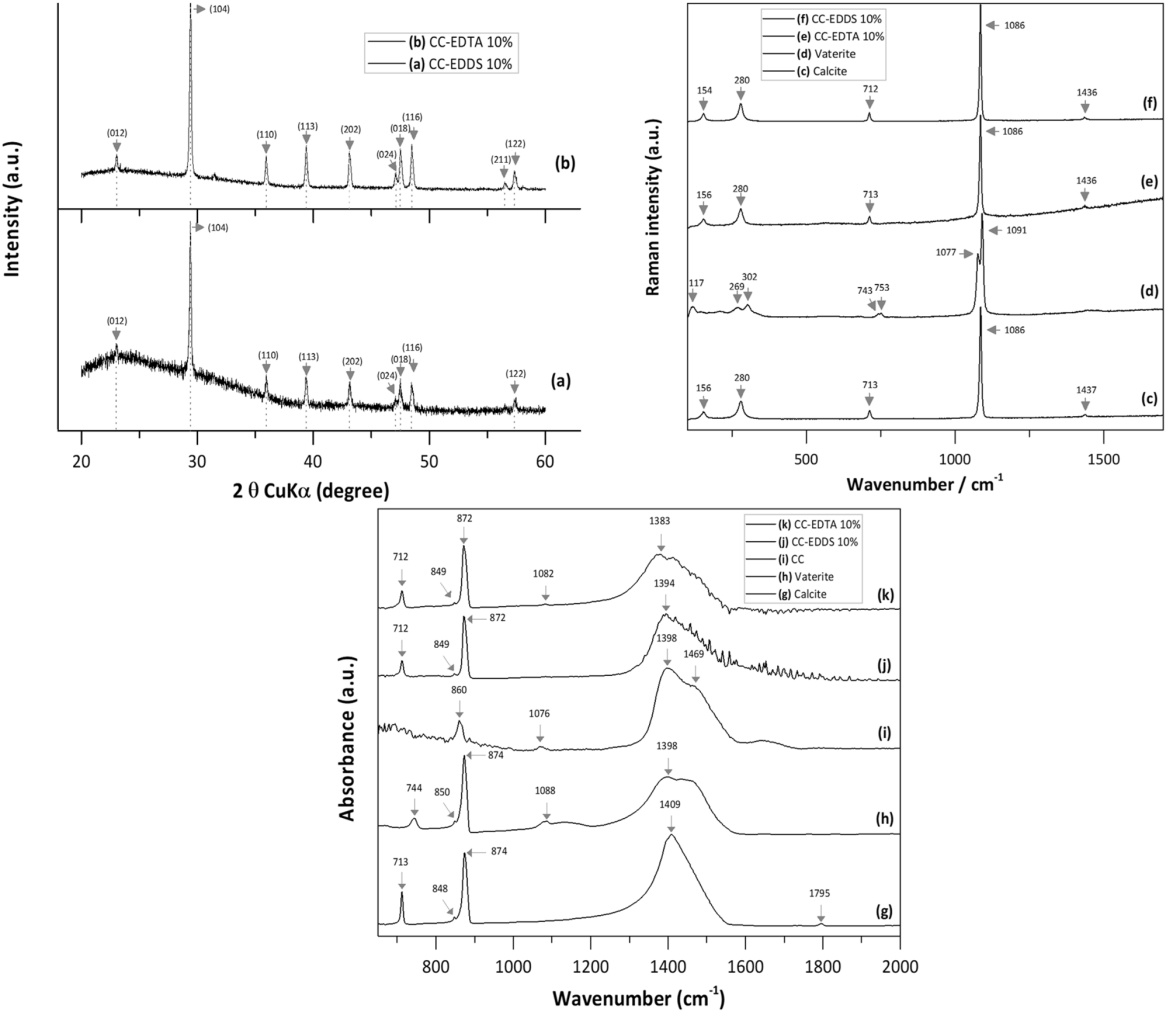
XRD, Raman and FTIR spectra of synthetic calcite.

The experimental XRD spectra obtained in the current study (Fig. 2) match well with the crystal structure of calcite^41^. The strongest 2θ peak at the value of 29.4° (Fig. 2a, b) represent the main peak of calcite and correspond to the characteristic reflections with d-spacing of 3.036 Å^42^, respectively to the crystallographic plane of (104)^43^. By examining the XRD spectra, it can be observed that the final product has a calcite crystalline structure in both type of environments, with EDTA 10% and EDDS 10%. The X-ray diffraction pattern with peaks at 2θ values of 48.45°, 48.5° and 56.48° corresponding to the d-spacing of 1.887 Å, 1.875 Å and 1.631 Å indicate the presence of vaterite, as minor impurity. Similar X-ray lines for synthetic vaterite were identified at 1.858 Å and 1.647 Å^42^. Some differences were observed between the XRD spectra for the carbonates with EDTA 10% and EDDS 10%. The spectrum of carbonates with EDDS 10% (Fig. 2a) has a lower quality, rather broad bands and higher background noise. This could suggest a low degree of crystallinity or a slightly amorphous composition compared to the spectrum of carbonates with EDTA 10% (Fig. 2b).

The Raman vibrational spectrum of calcium carbonates can be divided in three regions: the vibration of (CO_3_)^2−^ groups (internal modes), which represent the symmetric stretching ν_1_ and in plane bending ν_4_ of C–O bonds, and the region below 400 cm^−1^ that results from vibration between molecules in the lattice modes or external modes^44,45^. The experimental Raman investigations confirm in this study the presence of calcite and vaterite phases (Fig. 2c and d), which indicates that the amorphous state of CaCO_3_ could aggregate first in unstable vaterite before forming the crystalline state of calcite^46,47^. The calcite Raman features are also confirmed in the spectra of samples with 10% organic addition of EDTA and EDDS, as ACC stabilisers (Fig. 2e and f).

The synthetic calcite structures (Fig. 2c, e, f) showed the characteristic peaks^41^ with small differences between spectra in the absence and in the presence of 10% organic stabilisers. The most intense signals ν_1_ obtained at 1086 cm^−1^ are assigned to symmetric stretching of C-O bonds. These small differences of ν_1_ signals obtained and compare with others ν_1_ symmetric stretching at 1085 cm^−1^^45,48,49^ could be caused by the deviation in the lengths of C–O bonds in the disordered phase of ACC^44^. The Raman active lines attributed to ν_3_ mode (antisymmetric stretching) at 1436 cm^-1^ and 1437 cm^-1^, respectively are in good agreement with the literature^48^. Peaks at 712–713 cm^-1^ that corresponds to ν_3_ mode of C-O in-plane bending, are present in both synthetized products and calcite standard, confirms again the presence of calcite.

The Raman spectrum of vaterite (Fig. 2d) shows typical bands in the region between 1077 and 1091 cm^−1^ and correspond to the symmetric stretching vibrations ν_1_^45,46,49^ of (CO_3_)^2−^ groups. According to values reported in the literature^49^, the doublet ν_4_ in plane bending mode at 743–753 cm^-1^ it can be shifted to lower or higher wavenumbers. As vaterite is the most unstable CaCO_3_ polymorph after the ACC, and can be an intermediate precursor phase in calcite crystallization pathway, the presence of these vibrations in the Raman spectrum of the samples with 10% addition of EDTA, confirm the presence of vaterite phase and provide information about formation mechanism.

FTIR spectroscopy was also used for the characterization of synthetized calcium carbonate phases (Fig. 2g–k). The observed bands ν_2_ at 874 cm^−1^ and ν_4_ at 713 cm^−1^ (Fig. 2g) are characteristic for those reported in literature as featuring calcite^24,40,50^. CC spectrum (Fig. 2i) has a strong ν_2_ broad out of plane band at 860 cm^−1^ and a split peak attributed to ν_3_ (CO_3_)^2−^ asymmetric band at 1398 cm^−1^ and 1469 cm^−1^. The absence of the ν_4_ symmetric vibrations at 713 cm^−1^ or 744 cm^−1^ indicate that the calcium carbonate particles are amorphous^24,51^. In the vaterite spectrum (Fig. 2h) the absorption peak at 713 cm^−1^ is disappeared and a ν_4_ deformation band of (CO_3_)^2−^ is located at 744 cm^−1^^40,51,52^. Apparantly, according to FTIR results, the addition of 10% organics (Fig. 2j and k) does not have a significant influence in the formation of calcite phase. Besides that, the background noise could suggest an incipient phase in calcite formation. The FTIR spectra obtained are consistent with the observations from XRD and Raman results.

#### NTA

The nanoparticle tracking analysis (NTA) is a great method able to measure in real-time the particle size, dispersion, changes in the formation and stability of nanoparticles in different solvent systems^53^.

**Figure 3:**
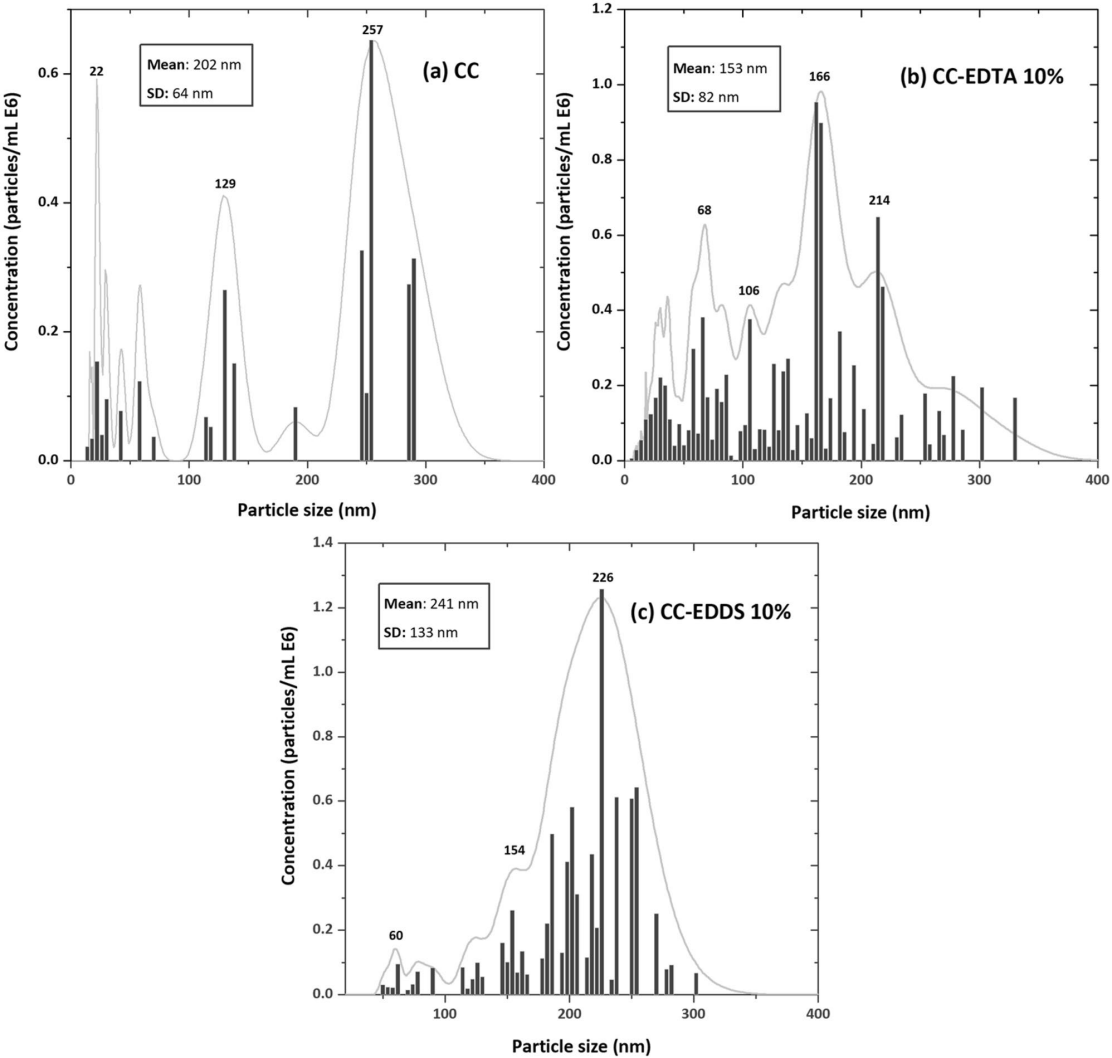
Hydrodynamic size of nanoparticles produced as standard precipitation of: (**a**) amorphous calcium carbonate (**b**) carbonates particles synthesis in addition with EDTA 10%, (**c**) carbonates particles synthesis in addition with EDDS 10%.

The obtained hydrodynamic size of nanoparticles characterized by NTA has shown that the particles are grouped in spheroidal clusters sizing between 200 and 300 nm (Fig. 3). The in situ and real time results from the aggregation in solution experiments show that after the first three seconds the nanoparticles are evidently separated in three individual molecular clusters with an average particle size of ca. 202 nm (Fig. 3a). From the NTA size distribution profile it can be distinguished three stages: a first stage of crystal nucleation up to 50 nm (indicative of the formation of amorphous calcium carbonate) that passed progressively through an intermediate stage up to 150 nm (that can be associated with the formation of vaterite) and a final stage of crystallization (most probably associated with the formation of calcite).

Comparing the results of the three systems, it can be seen that the addition of 10% organics in the synthesis lead to a decrease in the hydrodynamic size of the final particles for the EDTA system and an increase in the hydrodynamic size of the particles for the EDDS system, partially proving the initial hypotheses of the study (Fig. 3b and c), the role of EDTA and EDDS on nanoparticles scaffolding and their effect on surface properties.

#### BET

Surface area measurements for the CC plain particles gave values of 7.18 m^2^g^−1^ and 60.3 m^2^g^−1^ following the BET-Multiple points analysis method (BET-MPM) and the Langmuir method (LM), respectively. Further comparison will be carried out using BET-MPM values, as they are most popular and preferred values presented in the literature^54^.

The addition of 10% EDTA led to an increase of the surface area, up to 11.16 m^2^/g, whereas the addition of 10% EDDS led to a substantial decrease (ca. three times smaller) of the surface area, down to 2.32 m^2^/g. These differences will have an important impact on adsorption properties such as pollutants uptake capacities and pollutants removal efficiencies.

However, it is to be noted that the BET results (Table 1) are in very good agreement with and respect the same trends as the NTA and the SEM results: smaller particles led to higher surface area.

**Table 1:** Summary of BET surface properties of the particles used for adsorption experiments.

Material	Surface area (m^2^/g)	Pore volume CC g^−1^
CC	MPM = 7.18, R^2^ = 0.997 LM = 60.3	0.017
CC-EDTA 10%	MPM = 11.16, R^2^ = 0.998 LM = 362.5	0.049
CC-EDDS 10%	MPM = 2.32, R^2^ = 0.995 LM = 32.3	0.0049

Overall the BET, NTA and SEM results showed that the addition of EDTA in the synthesis protocol led to better surface properties from adsorption point of view (i.e., smaller particles, rougher surface, and bigger surface area and pore size) for the carbonates particles as opposed to the EDDS. The results show that the EDTA’s cis isomer, named EDDS, had an antagonist effect on CC scaffolding compared to the EDTA.

### RY84 adsorption onto CC

#### The effect of pH

Experimental adsorption studies were conducted at various pH values in order to evaluate the optimum uptake pH. The results are presented in Fig. S2. The trend of uptake capacity for the pH dependent experimental set (run at RT with C_RY84_ 3 mg/L and C_ads_ = 0.1 g/L) showed an increase from 27.99 mg/g at pH 6 to maximum of 65.83 mg/g at pH 8, after which it slightly decreases. The results might be related to the point of zero charge (PZC) of the carbonates and can indirectly provide some information about mechanisms that occurs during the adsorption. The ZPC for calcite was shown to lie within the range of pH 8–9.5^55,56^. It is known that surface charge of a solid at the pH of point of zero charge is null. At pH below the PZC the calcite surface is positively charged, whereas above it, the surface is negatively charged. As RY84 is a large molecule that contains functional groups that may charge differently in water as a function of pH: they can become positively charged (protonated, methyl, amino) as well as negatively charged (sulphate, chloride) (see Figs. S1 and S2), heaving a zwitterionic character. This can suggest that theoretically, the RY84 adsorption occurs predominantly between anionic sulphate or chloride ions and protonated surface of carbonates particles at pH below the pH of PZC and predominantly between negatively charged surface of carbonates particles and cationic amino and methyl groups of the RY at the pH above the pH of the PZC as well as potentially at pH of PZC via both types of functional groups, however competitively. Similar mechanistic hypotheses that were confirmed to a certain extent by the results were found in literature^57,58^.

As the optimum uptake capacity was found at pH 8 the following experiments were run at this pH.

#### Adsorption studies onto EDTA/EDDS functionalized CC

**Figure 4:**
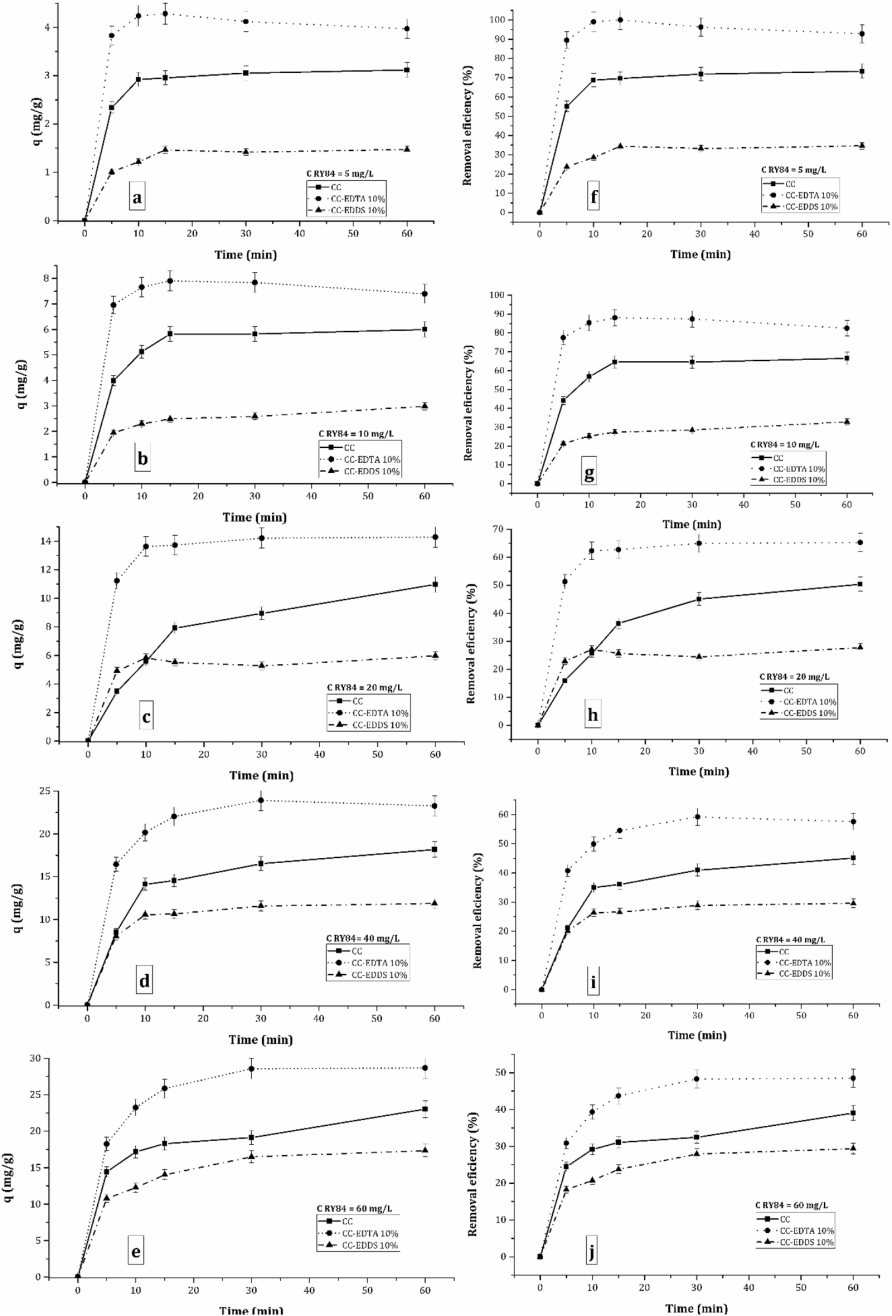
The kinetic profiles of the uptake capacity (q, left) versus removal efficiency (E, right) of RY84 adsorption onto ACC without and with addition of EDDS 10% and EDTA 10%. Experimental conditions: C_RY84_ = 5 to 60 mg/L, C_ads_ 1 g/L, batch adsorption at pH 8, T = RT, dynamic regime.

The kinetic profiles of the RY84 adsorption onto the three types of particles synthetized and varying the concentration of RY84 are presented in Fig. 4. The results are expressed as the uptake capacity of the adsorbent (Fig. 4a–e), as well as RY84 removal efficiency from the dye aqueous solution made in distilled water (Fig. 4f–j). First parameter is an expression of the adsorbent ability to load the pollutant under specified experimental conditions and considers adsorbent surface properties (such as surface area, surface charge and particle size), ultimately allowing comparison among the adsorbent’s performances. The second parameter, the removal efficiency, features waste quality by expressing the percentage of the pollutant up taken by the applied treatment, here adsorption.

From the kinetic profile of the adsorption it can be seen that the RY84 adsorption occurs relatively fast, the equilibrium is achieved at about 10–20 min, slightly increasing as the RY84 concentration increases. This trend is seen in the uptake capacity kinetic profiles as well as in the RY84 removal efficiency kinetic profiles.

Among the three systems corresponding to each type of the adsorbent used, it can be noticed that at all chosen concentrations the highest performance was achieved by the CC-EDTA 10% adsorbent followed by the CC and CC-EDDS 10%, despite of the initial RY84 concentrations used. The uptake capacity of the CC derived particles is ca. two thirds of the CC-EDTA 10%, while CC-EDDS 10% adsorbent takes up only half of the CC-EDTA 10% adsorbent.

This suggest that the presence of EDTA led to a significant increase of active surface sites available for RY84 binding, while, contrary, the EDDS reduced the surface sites available on the ACC transformation end product particles. These results are in good agreement with the BET measured surface properties of the three transformation products, which indicate a decrease of surface area as follows: CC-EDTA 10% > CC > CC-EDDS 10%.

Quantitatively Fig. 4a–e shows that the uptake capacity increased with increasing the RY84 concentration reaching the highest values of 28.7 mg/g for the CC-EDTA 10% system, 23.00 mg/g for the CC plain system and 17.34 mg/g the CC-EDDS 10% system, in which RY84 initial concentration was set at 60 mg/L. This coherent trend is nicely observed at all chosen concentrations, but it seems not to be linearly proportional with increasing RY84 concentration from concentrations above 10 mg/L, indicating that adsorbents surface may start approach saturation.

RY84 removal efficiency wise, it is noticed that 100% RY84 removal occurred at lowest RY84 concentration used, 5 mg/L, and it decreased to 48% at the highest RY84 concentration used (60 mg/L) (Fig. 4f–j). Again, although coherent, this decrease within the concentration interval studied, it was not linearly proportional with increasing concentration. These results suggest that for a contaminated water containing 5 mg/L RY84 an adsorbent dosage of 1 g/L is sufficient to ensure an efficient wastewater treatment, whereas for higher concentrations of RY84 in polluted wastewaters, the CC-EDTA 10% dosage should be adjusted in a non-linear proportional manner.

Although the kinetic profiles showed that the adsorption equilibrium has been reached in all systems, the conclusion that the surface saturation was reached in all systems it cannot be made. This can be seen by comparing q with E profiles at each working concentration. Thus, as example, focusing on the data from the experiment run at the middle concentration chosen, i. e., 20 mg/L, for the CC- EDTA 10% system (see Fig. 4c and h), the addition of 1 g/L adsorbent led to a maximum adsorbent load 14.2 mg/g that ensured an 65% efficiency of RY84 removal from solution. Further, by increasing RY84 concentration (to 40 mg/L, Fig. 4d and/or 60 mg/L, Fig. 4e) the adsorbent load increases (to 23.5 mg/g and 28.7 mg/g, respectively), suggesting that the adsorbent has still potential to uptake more RY84. Thus, those 35% of RY84 from solution at 20 mg/L could have been very well up taken. Moreover, by looking at q and E profiles for system at higher RY84 concentrations, it seems that the adsorbent did not reach the saturation under conditions above 20 mg/L, and that there are still RY84 molecules in solution to be up taken: ca. 45% and 55% for the 40 and 60 mg/L systems, respectively (the efficiency of RY84 removal being ca. 55% and 45% for the 40 and 60 mg/L systems, respectively). On another hand, a decrease of removal efficiency with the increasing RY84 concentration in solution that concomitantly led to an increase of adsorbent load may suggest that the RY84 binding is limited to only specific surface sites.

From the above interpretation of the results, it can be concluded that the adsorption is a complex interplay of mechanisms that occurs simultaneously to a specific extent. It is limitative not only by RY84 concentration or adsorbent dosage, surface charge/surface area, sites densities and their type but also the molecule size of RY84 that may only bind specifically at physically accessible sites.

Overall, from process engineering point of view, it is useful to display the results as q as well as E, as they provide important information about sorption mechanism as well as help process engineers dose the appropriate amounts of adsorbent to achieve targeted removal efficiencies.

As best quantitative results, among the three adsorbents chosen for the adsorption, were given by the CC doped with EDTA 10%, only these nanoparticles will be further treated for the kinetic and isothermal modelling and photodegradation studies.

#### Kinetic modelling

Adsorption kinetic modelling fits for the CC doped with EDTA 10% system are presented in Table 2. The kinetic modelling allowed the determination of weighted values for uptake capacities (at equilibrium, under specific experimental conditions that are mentioned in the table) as well as derive adsorption rates for each individual system. Fitted q values, that provides quantitative information about the RY84 uptake capacity at equilibrium can further be used in literature comparisons with other sorbents or other pollutants, but only if experimental conditions (pH, solid to liquid or pollutant to solid ratios, temperature), are similar. The goodness of fit is appreciated function of the highest value of adjusted regression coefficient—adjusted R^2^^59–62^. Considering the assumptions of each kinetic model are based to, a better fit to one of the models chosen provides indirect and empirical information about adsorption mechanism. Thus, generally a better fit to the PFO kinetic model implies that the adsorption mechanism occurs preponderantly via physical sorption, while a better fit to the PSO kinetic model implies a chemical sorption mechanism.

**Table 2:** Summary of the PFO and PSO kinetic modelling parameters for the CC-EDTA 10% system.

RY84 conc. (mg/L)	PFO kinetic model			PSO kinetic model		
	q (mg/g)	k_1_ (min^−1^)	Aj. R^2^	q (mg/g)	k_2_ (g mg^−1^ min^−1^)	Aj. R^2^
5	4.154 ± 0.065	0.521 ± 0.093	0.994	4.184 ± 0.06	0.838*	0.991
10	7.713 ± 0.102	0.465 ± 0.059	0.996	7.852 ± 0.11	0.256*	0.993
20	14.141 ± 0.099	0.315 ± 0.013	0.999	14.924 ± 0.316	0.046 ± 0.009	0.995
40	23.250 ± 0.391	0.229 ± 0.017	0.995	25.135 ± 0.5881	0.016 ± 0.002	0.995
60	28.261 ± 0.515	0.189 ± 0.014	0.995	31.081 ± 0.556	0.009 ± 0.001	0.997

Experimental conditions: C_RY84_ = 5–60 mg/L, batch adsorption at pH 8, T = RT, C_ads_ 1 g/L.

Modelling the adsorption experimental data for the set of the experiments, in which the RY84 concentration was varied from 5 to 60 mg/L, with the pseudo first order and pseudo second kinetic models, the following coherent trends regarding the weighted values of q and reaction rates were observed: the uptake capacity increases coherently (from ca. 4.154 to 28.261 mg/g) with the increase of RY84 concentration (from 5 to 60 mg/L) and the adsorption rate values decrease with increasing concentration (Fig. S3). These coherent trends visible on both models fits suggest that the adsorption occurs slower as RY84 concentration increases.

Looking at the regression coefficient, it can be noticed that the R^2^ values obtained for the PFO and the PSO are close to unit, slightly differing at the third decimal (Table 2). This fact suggests that, generally, adsorption take place via physical and chemical sorption mechanisms, interplaying to a specific extent as function of RY84 concentration. However, a closer look showed that at low RY84 concentrations (up to 20 mg/L) the data are slightly better fitted by the PFO kinetic model as opposed to the PSO model, suggestion that in these systems the adsorption mechanism is preponderantly physical sorption (occurring via H or Van der Walls bonding).

At RY84 concentration of 40 mg/L, the modelling fits returned an identic regression coefficient, fact that suggest that both mechanism, physical and chemical sorption occurs equally in the same system. Interestingly, this concentration seems to be the limit at which a slight change in the proportionality of the mechanisms change, by taking over a preponderantly chemisorption mechanism (that occurs via strong chemical bonding, i.e., covalent and coordination bonding).

#### Adsorption isotherms

The experimental data from experiments were RY84 concentration was varied (5–200 mg/L), but sorbent dosage was kept constant (1 g/L), led to plots of the adsorption isotherms. The data were fitted with the most common adsorption models such as Langmuir and Freundlich and their fits are displayed in Fig. S4 with the summary of the fitting parameter presented in Table 3. Both models are theoretical and/or empirical expression of adsorption equilibrium, and they involve various assumptions used to derive indirect mechanistic information. Moreover, they can be used to obtain and compare maximum adsorption capacities of various adsorbents for specific adsorbents or of various adsorbents for the same adsorbent, under similar or ideally identical process conditions^63–65^.

**Table 3:** Summary of fitting parameters from adsorption isotherms modelling with the Langmuir and Freundlich models.

	Langmuir isotherm	Freundlich isotherm
Parameters	q_max_ = 39.52 ± 2.94 mg g^−1^ b = 0.08 ± 0.02 R_L_ = 0.076–0.760	K_f_ = 6.685 ± 1.31 mg g^−1^ 1/n = 0.301 ± 0.03
Statistics	Adj. R^2^ = 0.961 Red. Χ^2^ = 6.4562	Adj. R^2^ = 0.956 Red. Χ^2^ = 7.568

Experimental conditions: C_RY84_ = 5–200 mg/L, batch adsorption at pH 8, T = RT, C_ads_ 1 g/L.

The modelling yielded a slightly better fit to the Langmuir isotherm (Adj. R^2^ = 0.961) compared to the Freunlich isotherm (Adj. R^2^ = 0.956) (Table 3 and Fig. S4). The best fit to the Langmuir model indirectly suggests that the adsorption may occur as a monolayer with a maximum up take capacity of 39.52 mg/g. The positive RL parameter, calculated from Langmuir parameters indicated that adsorption is favourable.

From the adsorbent point of view, the adsorption optimal experimental conditions for the CC-EDTA10% adsorbent are: max uptake capacity of 39.53 mg/g at pH 8, room temperature, dynamic regime and dye concentration of 60 ml/L. Efficiency wise, the optimal experimental conditions obtained are: 100% removal at 1 g/L adsorbent dosage and dye concentration of 5 mg.L under stirring condition at room temperature and pH 8.

The maximum uptake capacity obtained from isothermal fits allow comparison of the CC-EDTA10% performance with other adsorbents found in literature and used for RY84 uptake as well as helps process engineer guide for dosing the correct amount of sorbent into a specific wastewater system to ensure the targeted level of dye removal.

Presented results showed that the addition of EDTA provided superior uptake capacity for the CC as adsorbent as opposed to EDDS. There are no many adsorption studies on RY84 in literature. Similar RY dyes adsorbed onto a variety of bio and geo, natural and synthetic absorbents were found (Table S3). Thus, a sensible comparison is limited by the lack of adsorption studies for the RY84 azo dye. However, accounting on El Haddad et al., (2012)^66^, Barka et al., (2011)^67^ and Abdolmohammad-Zadeh et al.^68^ works on RY84 adsorption onto animal bone meal, hydroxyapatite and nano Zn-Al layered double hydroxide, respectively the maximum uptake capacity was compared (Table S3). The CC-EDTA 10% has very good uptake capacity for RY84 (39.53 mg/g) compared to other sorbents tested in the literature such as: hydroxyapatite (48.84 mg/g)^67^, nano Zn-Al layered double hydroxide (13.75 mg/g)^68^ and animal bone (57.15 mg/g)^66^ (see Table S3). Outstanding uptake capacities for RY84 were found for functionalised chitosan based adsorbents at pH 3–4. The authors have found maximum q values, of 2234.3 mg/g, 2000.1 mg/g and 1774.2 mg/g for RY84 onto chitosan with epichlorohydrin, ECH-CHs, chitosan with glutaraldehyde, ALD-CHs and Chitosan, CHs, respectively^69^. However, despite their great performance the adsorbents are made using compounds with a high degree of toxicity (epichlorohydrin is classified by several international health research agencies and groups as a probable or likely carcinogen in humans (stomach and lungs); glutaraldehyde is toxic and a strong irritant with potential high risk for cancerous). Thus, their use and final disposal post adsorption may pose environmental concerns due to their toxicity.

Differences of our results compared to the literature findings may be explained by differences in pH as well as adsorbent surface properties. Generally, the adsorption capacity strongly depends on adsorption surface properties (surface charge properties, size, surface area, pore presence and size, sites densities, the concentration of acidic/ wick acidic, basic functional groups etc.), sorbent stability under specific conditions (i.e. pH). pH influences the surface chemistry as well as pollutant chemistry in solution beside process controlled parameters such as mixing type and speed, rheology, temperature etc.). It is also observable that RY84 possesses a high molecular weight (1922.45 g/mol) as compared to other RY dyes (Fig. S1 and Table S3). On specific adsorbents surfaces it is possible that due to molecule size, its adsorption at adsorbent sites to be physically limited and thus led to a lower uptake capacity.

Thus, the necessity of coupling the adsorption process with the photodegradation process may rise in order to improve the RY84 removal efficiency at sites where its concentration in industrial effluents are elevated.

### UVA-induced photodegradation studies

**Figure 5:**
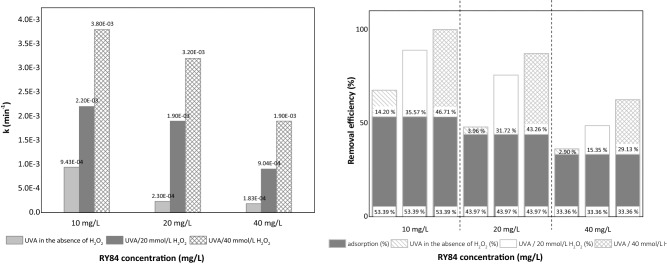
(**a**) Photodegradation rate constant (k, min^−1^) of RY84 after adsorption using CC-EDTA 10% nanoparticles (1 mg/L) at different concentrations in the absence and presence of H_2_O_2_. (**b**) Total removal of RY84 via consecutive adsorption and UVA/H_2_O_2_ processes. Experimental conditions: pH 7.3 RT, adsorption time 60 min (dynamic regime), photodegradation time 180 min (static regime), R^2^ is between 0.98 and 0.99.

The photodegradation process was investigated using the supernatant from the most efficient adsorbent, CC-EDTA 10%, under UVA irradiation. The initial concentration of RY84 solutions was set from 10 to 40 mg/L. It is notable that, when exposed to UVA light in absence of H_2_O_2_, a small conversion was observed (up to 14%, Table S4). In the presence of both H_2_O_2_ and UVA light the conversion increased substantially due to the hydroxyl radicals generated during the photochemical reactions. After 60 min of irradiation the removal of 10 mg/L RY84 were only 5% and 24% in absence and presence of H_2_O_2_, respectively. After 180 min of irradiation the removal increased to 14% and 49% in absence and presence of H_2_O_2_, respectively, whereas only 2.90% and 29% of 40 mg/L RY84 were removed, respectively. The corresponding (pseudo) first order constants were 9.43 × 10^–4^ min^−1^ for 10 mg/L RY84 in absence of hydrogen peroxide and 3.8 × 10^–3^ min^−1^ in presence of H_2_O_2_ (Fig. 5a)_._ The photodegradation rate constant increased as the initial concentration of RY84 is decreased. The lower removal of RY84 degradation at higher initial concentration of dye is probably due to absorption of light at 365 nm and competition for hydroxyl radicals by compound. The same inhibiting tendency was observed for degradation of different pollutants^70–72^. The pseudo first order constant in the presence of H_2_O_2_ (3.8 × 10^–3^ min^-1^) for the removal of 10 mg/L RY84 is over four times greater than that with no H_2_O_2_ (Fig. 5a). From practical point of view this means that the volume of the photoreactor for the same flow rate of water can be over four times smaller.

### Removal of RY84 from wastewater effluent by adsorption and photodegradation processes

**Figure 6:**
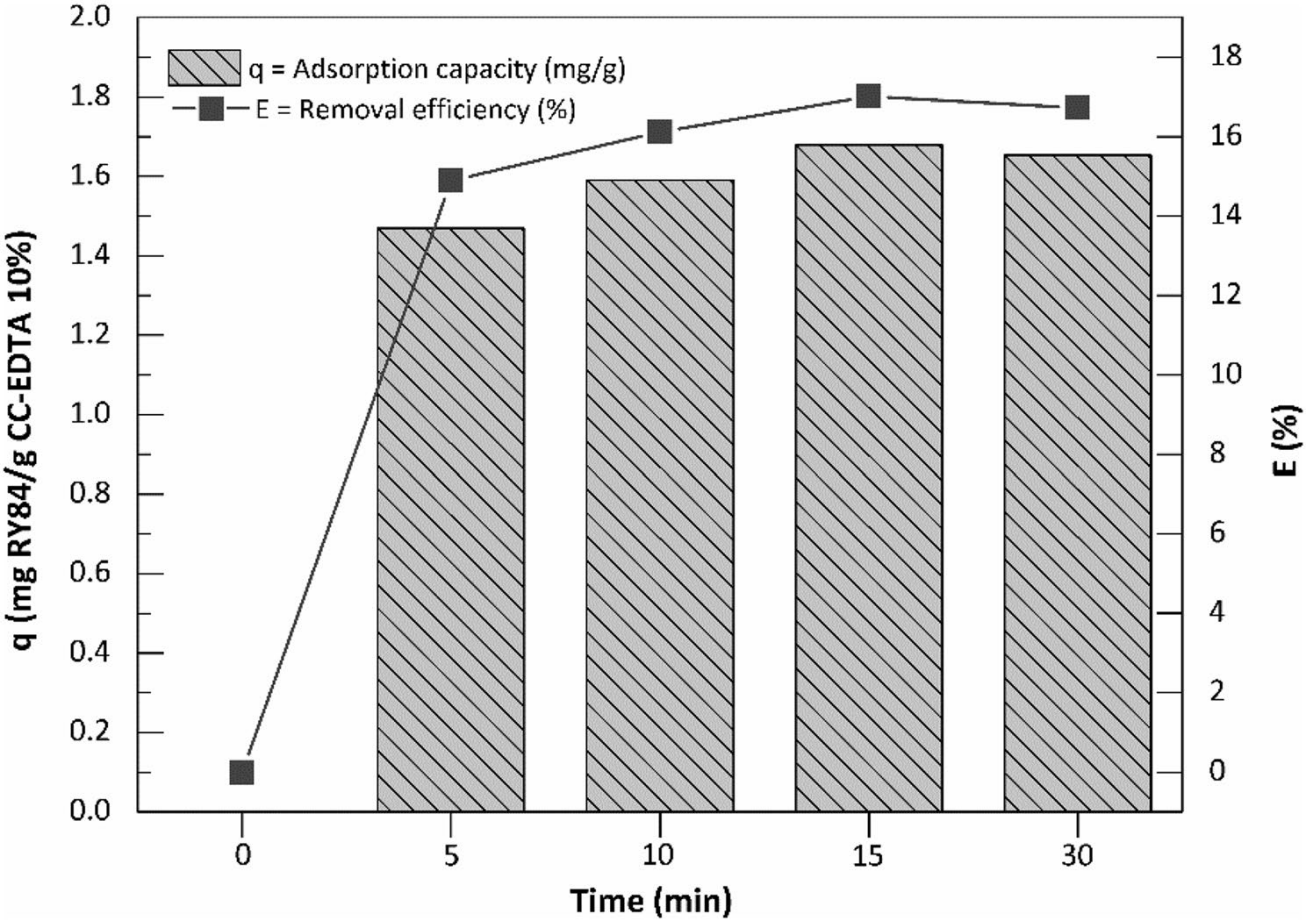
The kinetic profiles of the uptake capacity versus removal efficiency of RY84 adsorption onto CC-EDTA 10% in wastewater.

The experimental results obtained from testing the uptake capacity of CC-EDTA 10% for RY84 removal from real wastewater are presented in Fig. 6. A comparison of the adsorption results obtained from the experiments run in distilled water vs. wastewater at the same RY84 concentration (10 mg/L, Fig. 4), show that the adsorption occurred fast but with substantial decrease (ca 4 times) of the adsorption capacity (from ca. 7.7 to 1.8 mg/g) as well as RY84 removal efficiency (from 88 to 18%).

The decrease can be explained by competitive effect of other ions present in the wastewater background that bind preferentially at adsorbent surface sites and led to ca. 75% occupancy of the surface sites. This output, is essential for industrial process designing as it can guide the process engineer to consider significant adjustments in the adsorption dosage during the adsorption process, to reach the targeted efficiency of the RY84 removal. Thus, we performed the removal of RY84 using two consecutive processes: photodegradation after adsorption to obtain simulations of removal processes that might be used during water treatment in textile industry.

**Figure 7:**
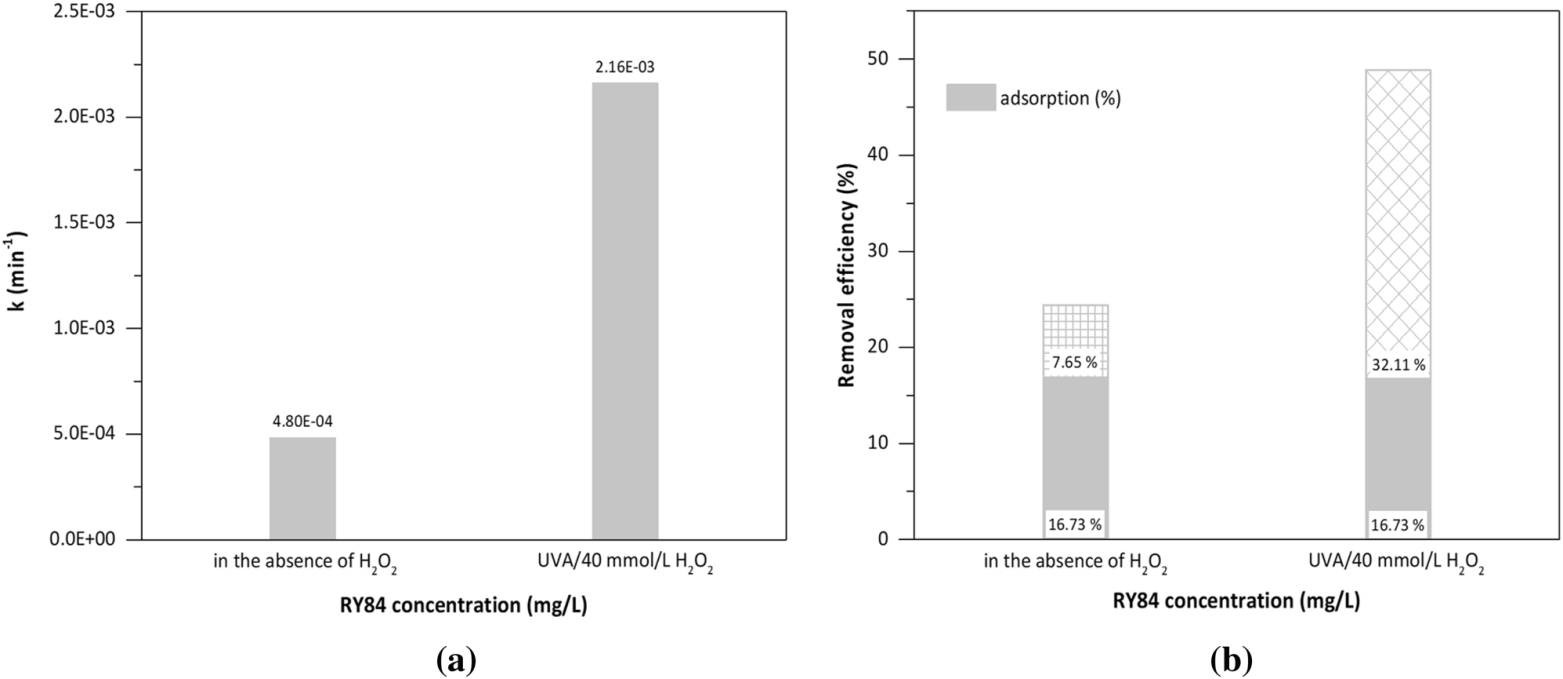
(**a**) Photodegradation rate constant (k, min^−1^) of RY84 after adsorption using CC-EDTA 10% nanoparticles (1 mg/L) in the absence and presence of H_2_O_2_. (**b**) Total removal of RY84 in wastewater effluent by adsorption and photodegradation processes. Experimental conditions: pH 7.3 RT, adsorption time 60 min (dynamic regime), photodegradation time 180 min (static regime).

The efficiency of the RY84 photodegradation in wastewater in the presence of 20 and 40 mmol/L of H_2_O_2_ decreased (ca. five times) compared to the degradation efficiency in the distilled water (from 35.57 to 7.65% and from 49.11 to 32.11%, respectively) (Figs. 5b and 7b). The photodegradation of RY84 in wastewater effluent was slower (k = 4.8 × 10^–4^ min^-1^) than in the experiments with dissolved natural organic matter (DNOM)-free water (MilliQ water) (k = 9.43 × 10^–4^ min^-1^) (Fig. 7a and Table S5).

This can be explained by the effect of common wastewater constituents such as HCO_3_^−^, NO_3_^−^, total suspended matter and Fe (III) ions on photodegradation of RY84, probably due to absorption of light and competition for hydroxyl radicals by existing scavengers in water (humic-like substances). It is generally accepted that DNOM effects the degradation of pollutants dissolved in water through various reactions. In most cases, the natural organic matter quenches the excited state of the pollutant. Our results are in agreement with the data reported by Rueda-Marquez^73^, who found that the removal of organic pollutants strongly decreases in the presence of complex real wastewaters.

Overall, the current results have important implications to wastewater treatments in textile industry and appropriate decisions making, from many respects, including: (1) the choice of treatment process, (2) wastewater treatment design, (3) control and monitoring of the processes from the point of view of process engineering, as well as (4) further process optimization and scaling up to pilot and industrial levels. Coupling the two processes in wastewater treatments can be a feasible alternative at sites where the concentration of refractive reactive dyes in wastewater is high and where only one of the removal processes would prove to be an insufficient. Although the photodegradation has the advantage that it totally or partially degrade the RY84, for large molecules this process is relatively slow, thus an adsorption step could be used prior photodegradation to minimise the content of dye. On the other hand, the adsorption process has the advantage that concentrate most of the dye fast on an environmentally friendly sorbent, that can be regenerated by thermal treatments and reuse in multiple cycles^74^. This will make the subject of further research.

## Methods

### Materials

Pure grade chemicals such as CaCl_2_ (CAS: 10043–52-4), Na_2_CO_3_ (CAS: 497–19-8), NaOH (CAS: 1310–73-2), HCl (CAS: 7647–01-0), EDTA (CAS: 139–33-3), EDDS (CAS) (Fig. S1) and distilled deionized water were used for materials synthesis. These analytical high purity reagents were purchased from Sigma Aldrich. The adsorption proprieties were investigated by using Reactive Yellow 84 (RY84) as model dye.

### Experimental setup

The synthesis experiments were carried out by mixing equal volumes of equal concentrations of 100 mmol/L Na_2_CO_3_ and 100 mmol/L CaCl_2_ under rigorous stirring. The addition of 10% EDTA and EDDS was made in Na_2_CO_3_ solution before mixing with the CaCl_2_ solution. The pH was checked and adjusted when needed to a constant value of 11.2 with 2 mol/L NaOH or 10 mmol/L HCl.

### Analytical methods

A HITACHI S-3400 N Type II Scanning electron microscopy (SEM) was used to collect information about materials morphology and size. Micrographs were collected at different magnifications (500–10 k) at a voltage set to 18–20 keV and a working distance set at 4.3 mm. The crystalline composition and structure of the calcium carbonate phases were characterized by X-ray diffraction (XRD, Shimadzu X-ray diffractometer) using Cu-Kα radiation at 40 kV and 30 mA. The diffraction pattern in the 10–80° 2θ range was collected at a scanning angle step of 0.02° and 2°/min^-1^ scan speed. Evaluation of diffraction patterns and identification of the carbonates polymorphs present in final materials was performed using the Calcite and Vaterite databases^41,43^.

Raman spectra were acquired using a Renishaw in Via-Raman microscope equipped with a diode laser of 785 nm (or and 473 nm). A beam size of 5 ×, 20 ×, 50 × and 100 × long working distance objectives was used to characterize the materials at the Diamond Light Source. The spectra were recorded at 50 s or 100 s acquisition time over the range 100–2000 cm^-1^.

A Micro Fourier Transform Infrared (FTIR) bench top spectrometer (Nicolet 6700 Thermo equipped with a Smart Orbit ATR and a diamond window) was used to collect infrared spectra Lab at the Diamond Light Source. Vibration frequency changes in carbonates materials were recorded with 64 scans per FTIR absorption spectra in the mid-infrared range from 4000 to 600 cm^−1^ at a resolution of 2 cm^−1^. Synthetic standards mixed with boron nitride were also used as FTIR standards. Particle size characterization was performed by NanoSight LM 20 device equipped with a laser beam that is introduced to the sample through a glass prism. Samples were measurement at a controlled temperature of 23 °C, manual camera shutter at 500, screen gain adjustment at 100–150 and a capture duration of 20 s. The samples were advanced between each recording to ensure replicate measurements. The NTA acquisition settings were optimized to track and measured each particle on frame-by-frame. The sample prism was cleaned after each recording video by using Milli-Q water until no visible particles were observed in the viewing windows. The performance of the system was investigated by measuring nanoparticle polystyrene latex microspheres of 400 nm. The BET adsorption measurements at − 196 °C (in liquid nitrogen) were performed on a NOVA 2200 Quantachrome instrument (Quantachrome Corporation, Boynton Beach, FL, USA). Samples were outgassed under high vacuum at room temperature (according to IUPAC and to ISO 9277) before physisorption measurements, so that physically adsorbed species were removed from the adsorbent surface. A dedicated software of instrument was used for the data fitting within the usual adsorption models (Brunauer–Emmet–Teller (BET) model) for specific area determination. The total pore volume was estimated directly from the nitrogen adsorption–desorption isotherm at relative pressure of P/P0 = 0.95.

### Adsorption experiments

Adsorption studies were conducted under dynamic regime assured by a magnetic stirrer at 150 rpm. The pH was measured and maintained constant during the all the adsorption experiments by the potentiometric titrator (SI Analytics7000) set on pH–stat method to keep the pH constant during the entire adsorption time set at 120 min. The titrator automatically added acid HCl (10 mmol/L) during the experiments at pH 5 and 7 and base NaOH (10 mmol/L) for the experiment at pH 9, in fine volume steps (0.01 mL), as required to maintain the pH constant. Experimental sets were run at RY84 initial concentration set from 5 to 60 mg/L, pH set from 6 to 11.2 and solid concentration of 1 g/L. The 3 mL aliquots were taken at various time intervals, filtered via 0.22 µm cellulose nitrate membrane and prepared for RY84 analysis by UV–Vis (Thermo Scientific Microplate reader Multiskan GO with cuvette) at ʎ = 400 nm. The UV–Vis detection limit for RY84 is < 1 mg/L. Adsorption experiments were run in duplicates. Blanks for pH and concentration dependent experiments were used to check materials dissolution. Standard deviation of the results was found below 5%.

### Photodegradation experiments

Photodegradation experiments in the presence and the absence of H_2_O_2_ were carried out in petri dishes (40 ml) and using a UVA bench lamp at 15 watts, 365 nm and 115 VAC/60 Hz (Cole-Parmer). Initial concentrations of RY84 were 10, 20 and 40 mg/L and exposure time was set to 3 h. After 60 min of equilibration, the adsorbent was removed by filtration using 0.2 µm filter and a solution of diluted 30% H_2_O_2_ was added to achieve the selected H_2_O_2_ concentrations (20 and 40 mmol/L), these being the basic conditions for experimental tests. Samples of the reaction medium were withdrawn at regular intervals for pollutant absorbance measurement. The kinetic of dye photodegradation in the presence as well as in the absence of H_2_O_2_, beside UVA, was fitted using first order kinetic model.

### RY84 uptake from wastewater via adsorption and photodegradation

Adsorption studies were also carried out by using wastewater. They were designed similar to distilled water experiments: room temperature and 150 rpm mixing speed, but only using one dye concentration and adsorbent dose (1 g/L). The wastewater was collected from municipal waste water treatment plant (WWTP) and spiked with minor volume of dye stock solution to obtain dye concentration of 10 mg/L. The initial pH of the dye polluted wastewater was 7.5. Photodegradation experiments post adsorption using wastewater were run in a similar manner as the ones run in distilled water. The characterization of wastewater is presented in Table S2.

## Supplementary Information

Supplementary Information 1.

## Data Availability

All data needed to evaluate the conclusion in this paper are presented in the paper.
