# Supplementary material for: Advanced removal of Reactive Yellow 84 azo dye using functionalised amorphous calcium carbonates as adsorbent
Source: Sci Rep. 2022 Feb 24;12:3112. doi: 10.1038/s41598-022-07134-2 (PMC8873491; doi:10.1038/s41598-022-07134-2)
Supplement: Supplementary file 1 — Supplementary Information 1. [file 41598_2022_7134_MOESM1_ESM.pdf]

Advanced removal of Reactive Yellow 84 azo dye using functionalised amorphous calcium carbonates as adsorbent

Loredana Brinza [a,*], *Andreea Elena Maftei* [a], *Sorin Tascu* [b], *Florin Brinza* [c], *Mariana Neamtu* [a,*]

[a] Department of Exact Sciences and Natural Sciences, Institute of Interdisciplinary Research, Alexandru Ioan Cuza University of Iasi, 700506 Iasi, Romania

[b] Research Center on Advanced Materials and Technologies, Department of Exact and Natural Science, Institute of Interdisciplinary Research, Alexandru Ioan Cuza University of Iasi, Blvd. Carol I, no. 11, 700506 Iasi, Romania

[c] Faculty of Physics, Alexandru Ioan Cuza University of Iasi, 700506 Iasi, Romania

Supplementary information

----

* Correspondence to: E-mail addresses: loredana.brinza@uaic.ro, mariana.neamtu@uaic.ro

Adsorption kinetics modelling

The best fit to one of the kinetic models would enable adsorption rates and weighted adsorption maximum uptake capacity to be compared under various conditions and also offer empirical mechanistic information about adsorption. Briefly here, a best fit to PFO kinetic model implies a mainly physical adsorption mechanism (based on weak interactions such as hydrogen or Van der Walls bonds) whereas a best fit to PSO kinetic model would indicate a chemical sorption mechanism (based on strong covalent bonds) (Table S1). It is also accepted that the PFO kinetic model assumes that the rate of pollutant sorption is proportional to the number of vacant sites onto the biosorbent, whereas the PSO kinetic model assumes that the rate of pollutant sorption is proportional to the square of the number of vacant sites on the biosorbent (*1, 2*).

Table S1. *Kinetic models parameters*

Kinetic model	Equation
**Pseudo first order model**	Non linear form: $\frac{dq_t}{dt} = k_1 \times (q_e - q_t)$<br>Linear form: $\ln(q_e - q_t) = \ln q_e - k_1 \times t$<br>$k_1 = pseudo\ first\ order\ kinetic\ rate\ cofficient, min^{-1}$
**Pseudo second order model**	Non linear form: $\frac{dq_t}{dt} = k_2 \times (q_e - q_t)^2$<br>Linear form: $\frac{1}{q_t} = \frac{1}{k_2 \times q_e{}^2} + \frac{1}{q_e} \times \mathrm{t}$<br>$k_2 = pseudo\ second\ order\ kinetic\ rate\ cofficient, g\ mg^{-1}min^{-1}$

Adsorption isotherms

Batch systems were used for the adsorption experiments designed for deriving the isotherms using 1g/L of sorbent concentration and concentrations of RY84 between 5-200 mg/L, at pH 8. The adsorption isotherms data were plotted as uptake capacities versus RY84 concentration at equilibrium. The modelling was performed by using Langmuir (*3*) and Freundlich models (*4*-*6*).

Langmuir model is a theoretical model which assumes that adsorption sites are homogenous distributed on adsorbent surface, are energetically similar and that the adsorbate adsorption takes place as monolayers. It accounts on the surface coverage by balancing the relative rates of adsorption and desorption (dynamic equilibrium). Adsorption is proportional to the fraction of the surface of the adsorbent that is open while desorption is proportional to the fraction of the adsorbent surface that is covered (*7*).

$$q_e = \frac{q_{max} b C_e}{1 + bC_e}$$

Where q_{max} is the maximum adsorption capacity (mg/g), q_e is the adsorption capacity at equilibrium (mg/g), C_e is metal concentration in solution at equilibrium (mg/L) and b is dimensionless Langmuir constant related to the feasibility of adsorption.

Derived from Langmuir isotherms is a so-called separation factor R_L which can be calculated by the following equation.

$$R_L = 1/(1 + bC_o)$$

Where *b* is Langmuir constant and *Co* is adsorbate initial concentration (mg/L).

The values of R_L assumes the nature and the feasibility of adsorption process as presented in table below:

R_L value	Adsorption process
$R_L > 1$	Unfavourable
$R_L = 1$	Linear
$0 < R_L < 1$	Favourable
$R_L = 0$	Irreversible

Freundlich isotherm is an empiric model which assumes that the adsorption sites are heterogeneously distributed and that the adsorption takes place as multilayer. It gives an expression which defines the surface heterogeneity and the exponential distribution of active sites and their energies (*7*).

$$q_e = K_f C_e^{1/n}$$

Where q_e is the adsorption capacity at equilibrium (mg/g), C_e is metal concentration in solution at equilibrium (mg/L), *Kf* is Freundlich constant (mg/g), 1/n is Freundlich exponent related to the adsorption intensity and it also indicates the relative distribution of the energy and the heterogeneity of the adsorbate sites.

**ACC**	**Complexing agents**	**RY84**
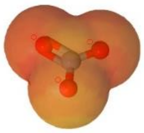<br>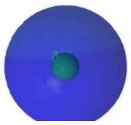	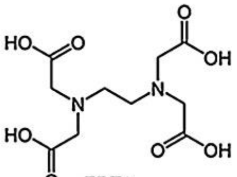<br><br>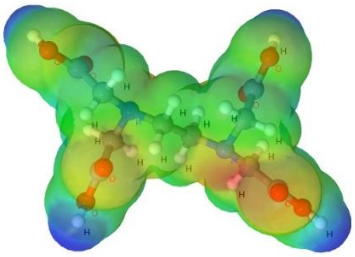	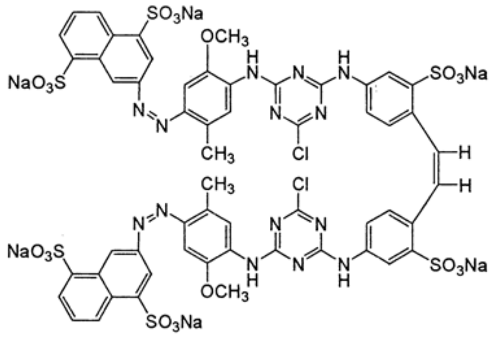<br>Main characteristics of the reactive yellow 84 used<br>Colour index RY84<br>Trade name Procion Gelb H-E4R<br>Chemical formula $C_{50}H_{24}Cl_2N_{14}\ Na_{10}O_{30}S_{10}$<br>Molecular mass (g/mol) 1,922.45<br>Water solubility at 293 K (g/l) 70<br>Phitotoxicity LC50 (mg/l) >100<br>lmax (nm) 406<br>Supplier BASF AG<br>Purity (%) 80.90
	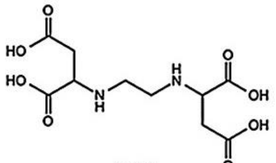<br><br>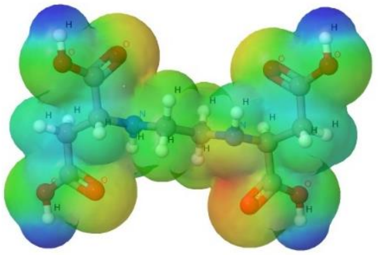<br>Stick and ball colour legend: H= white, O = red, C= light blue, N= green	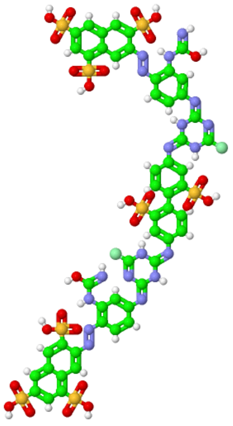<br>Stick and ball colour legend: C= green, S=yellow, H/Na= white, N= purple, Cl=light green

Fig. S1 *Main characteristics of materials used in adsorption and degradation experiments*

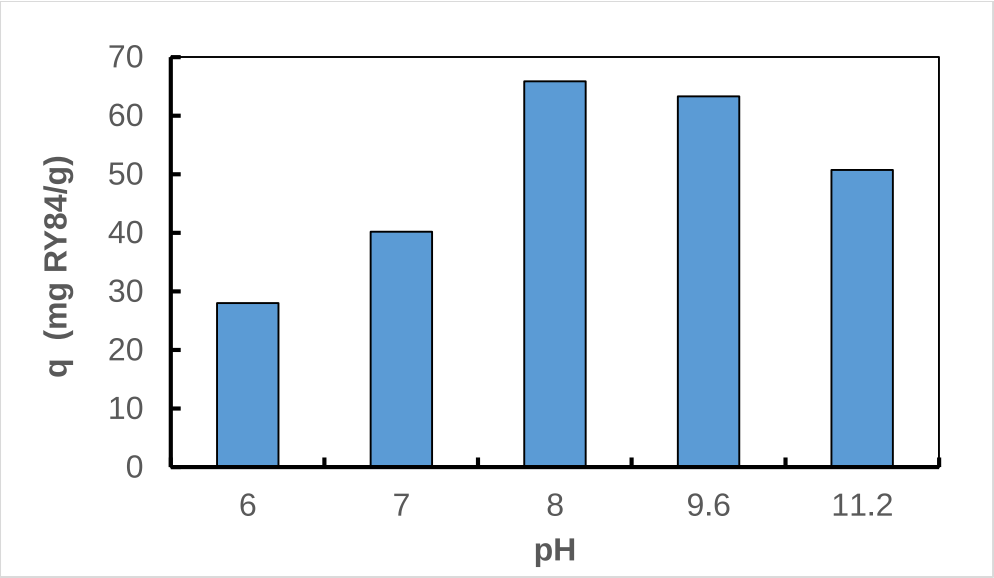

Fig. S2 *The profile of the uptake capacity of RY84 adsorption onto CC as a function of pH*

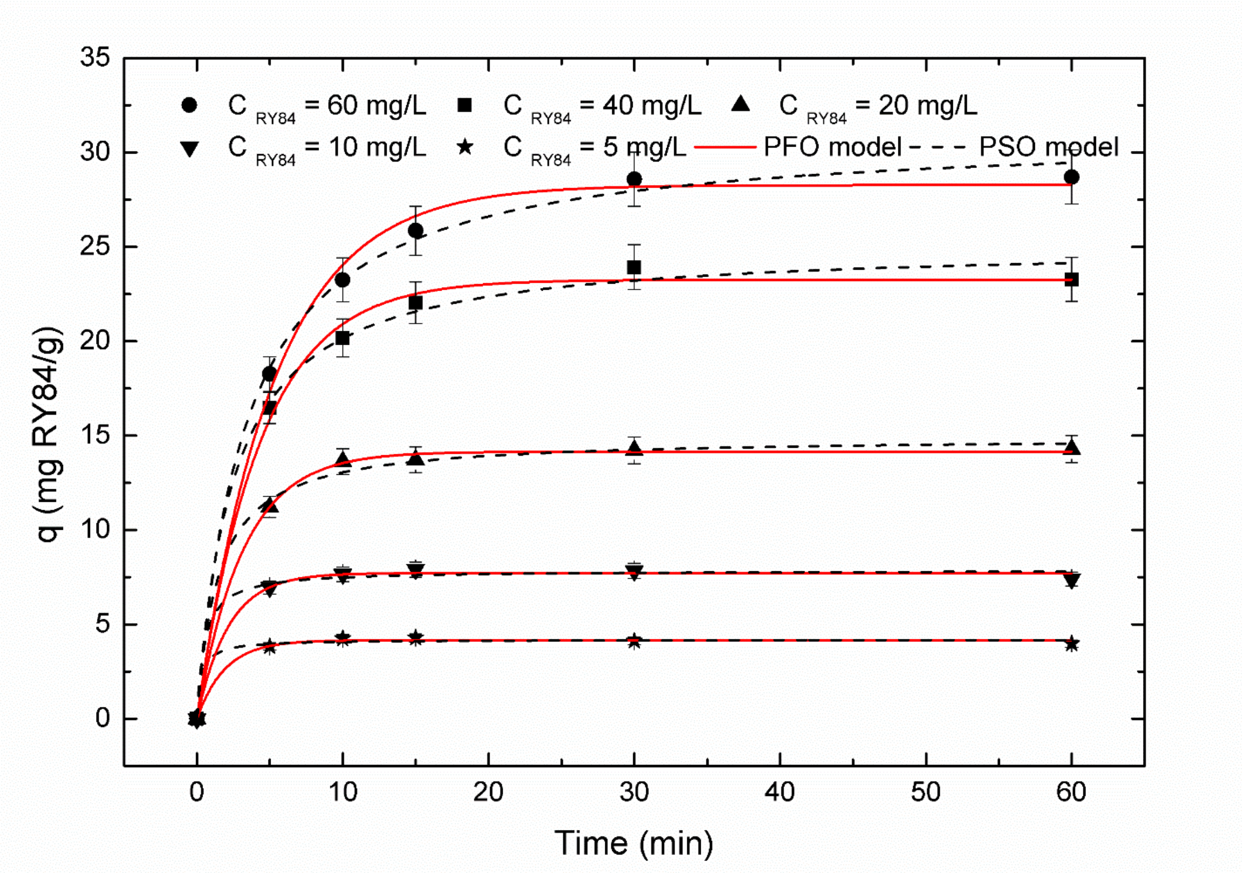

Fig. S3 *Kinetic profiles of RY84 adsorpion modeleld by pseudo-first order (PFO) and pseudo-second order(PSO) kinetic models.*

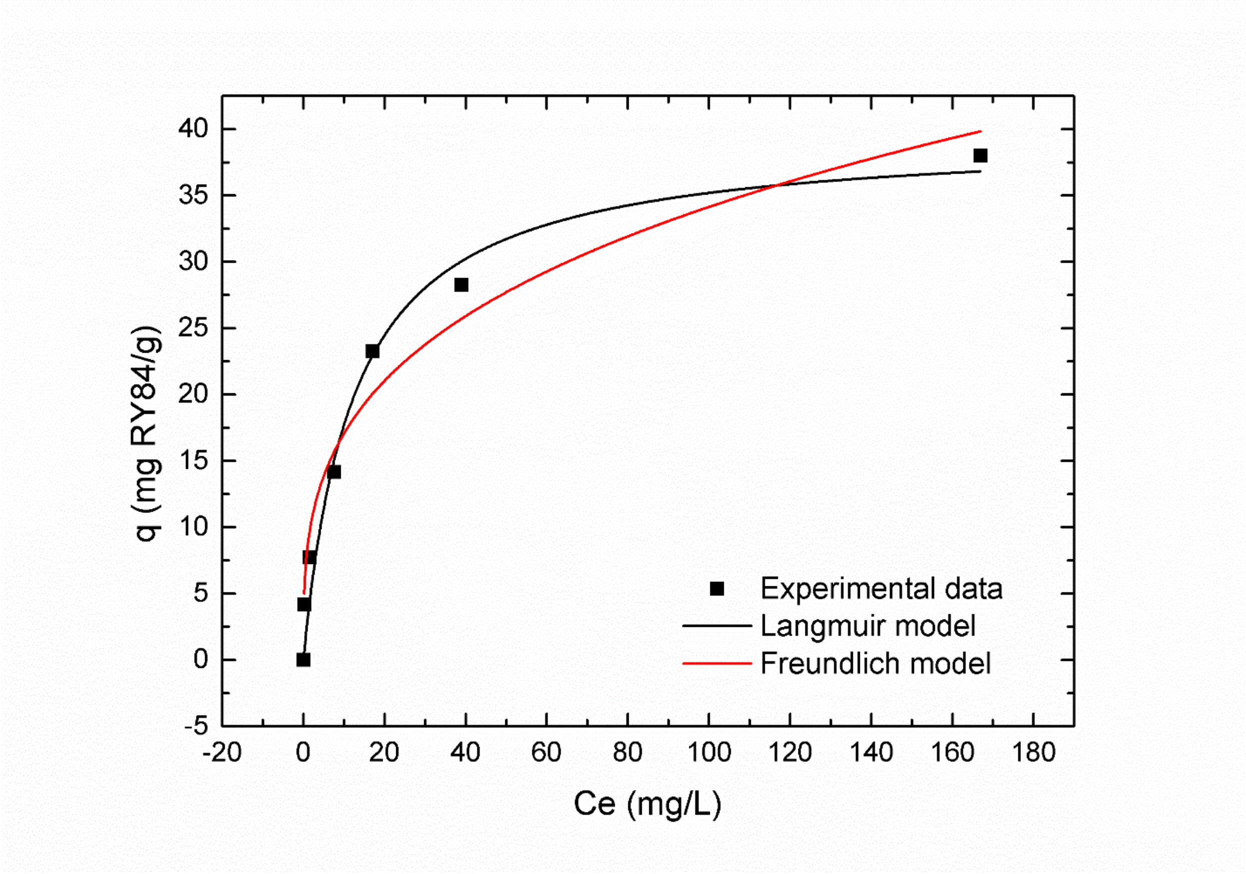

Fig. S4 *RY84 Adsorption isotherms modelling with the Langmuir and Freundlich models*

Table S2. *Chemical and physical parameters of non-diluted wastewater effluent from the WWTP*

Parameter	Unit	Value	Parameter	Unit	Value
**pH**	unit. pH	7.30	**Sulphides**	mg/L	0.05
**BOD5**	mgO_2/L	12.00	**TSP**	mg/L	12.00
**COD**	mgO_2/L	27.00	**Fixed residue**	mg/L	432
**NH_4^+**	mg/L	0.060	**Total N**	mg/L	8.40
**NO_2**	mg/L	0.050	**Total P**	mg/L	1.10
**NO_3**	mg/L	35.62	**Conductivity**	µS/cm	615

**Table S3.** *Comparison of RY dyes uptake capacity by others sorbents*				
**Dye - adsorbent details**	**q max, mg/g**<br>**mmol/g**	**Dye molecular weight**	**Process conditions**	**References**
RY3 onto modified palm leaf sheath fibers	83.19<br>0.14	592.99	pH 2	Dai et al 2021 (*8*)
RY15 onto nanomagnetite biocomposite from yeast biomass and magnetic nanoparticles	2.5<br>0.0039	634.6	pH 7	Nascimento, et al 2021(*9*)
RY64 onto calcinate alunite	236<br>0.640	368.2	Alkaline pH	Ozacar et al 2003(*10*)
RY81 onto multi walled carbon nanotubes	33.85<br>0.020	1632.18	pH 2 and 12	De Benedeto 2020 (*11*)
RY81 onto biochar synthesized from marine seaweed, Caulerpa scalpelliofrmis	151.5<br>0.092	1632.18	pH 2	Sundar et al 2020(*12*)
RY84 onto animal bone meal	57.15<br>0.029*	1922.45*	pH 6.6	El Haddad et al 2012(*13*)
RY84 onto hydroxyapatite	48.84<br>0.025*	1922.45*	pH 5	Barka et al 2011 (*14*)
RY84 onto nano Zn-Al layered double hydroxide	13.75<br>0.007*	1922.45*	pH 8	Abdolmohammad-Zadeh et al 2013 (*15*)
RY84 onto CC-EDTA10%	39.35<br>0.02	1922.45	pH 8	This study
RY86 onto silver nanoparticle-colemanite ore waste	68.71<br>0.103	667.357	pH 2	Yola et al 2014 (*16*)
RY84 onto chemically-cross-linked chitosan				
Chitosan with epichlorohydrin, ECH-CHs	2234.3<br>1.31	1701	pH 3	Filipkowska, and Jóźwiak 2013 (*17*)
Chitosan, CHs	1774.2<br>1.04		pH 4	
Chitosan with glutaraldehyde, ALD-CHs	2000.1<br>1.17		pH 3	
RY145 onto soy bean	87<br>0.084	1026.3	pH 2	Giordano et al 2021 (*18*)

**Table S3.** *Comparison of RY dyes uptake capacity by others sorbents*				
**Dye - adsorbent details**	**q max, mg/g mmol/g**	**Dye molecular weight**	**Process conditions**	**References**
RY160 onto natural clay modified by cocamidopropyl betaine (CAPB)	54	818.12	pH 2	Ghafar et al 2020(*19*)
RY onto Chlorela vurgaris Pristine CA modified	50.89<br>0.049	1026.3	pH 5	Radwan et al 2020 (*20*)
RY145 onto pumpkin seed	115<br>0.112	1026.3	pH 3	Benkaddour et al 2018 (*21*)
RY145 onto pine needle	7.225<br>0.0070	1026.3	pH 2	Ucar 2014 (*22*)
RY145 onto eggshell waste	88,45<br>0.086	1026.3	pH 2-5	Ofudje et al 2021(*23*)
RY145 onto chitosan	132.39<br>0.129	1026.3	pH 4	Karmaker et al 2015 (*24*)
RY145 onto textile sludge based activated carbon	96.8/<br>0.093	1026.3	pH 3	Oke et al 2022 (*25*)
RY145 onto TiO2, ZnO, and CTAB-stabilized Fe_3O_4 nanocomposite	212.76/<br>0.207<br>192.3/ 0.187<br>333.33/0.324	1026.3	pH 2	Rezaei-Aghdam et al 2021
Activated carbon	9.74<br>0.0094	1026.3	pH 5.5	Lafta et al 2015(*26*)
RY160 onto magnetite nanoparticles coated with CMK-8 ordered mesoporous carbon	62.893<br>0.076	818.12	pH 3	Toutounchi et al 2019(*27*)
RY160 onto natural glauconite, thermal activated glauconite and acid activated glauc.	29.85/0.036<br>51.55/0.063<br>55.87/0.063	818.12	pH 1	Younes et al 2021 (*28*)
RYX-RG onto Cucurbit (CB[8])	2135.4<br>3.252	656.39	pH na ultrasonic mixing for 15 min at RT	Li et al 2017 (*29*)
RYX-RG onto cucurbit (CB[6])	289.1<br>0.451	656.39	pH na ultrasonic mixing for 15 min at RT	Li et al 2017 (*29*)

**Table S3.** *Comparison of RY dyes uptake capacity by others sorbents*				
**Dye - adsorbent details**	**q max, mg/g mmol/g**	**Dye molecular weight**	**Process conditions**	**References**
RY GR onto silicates	351	634.6	Na	Moscofian et al 2013(*30*)
* As the molecular weight of the RY84 may vary slightly as function of its purity and supplier, the literature results were normalized using RY84 molecular weight of 1922.45 g/mol, because the source article does not state the appropriate molecular weight of the RY84 used.				

Table S4. *Photodegradation of RY84 (% of removal) at different initial concentrations (initial pH 8, concentration of H_2O_2 = 20 and 40 mmol/L)*

	**10 mg RY84/L**			**20 mg RY84/L**			**40 mg RY84/L**		
**Time min**	*UVA*	*UVA/20 mmol/L H_2O_2*	*UVA/40 mmol/L H_2O_2*	*UVA*	*UVA/20 mmol/L H_2O_2*	*UVA/40 mmol/L H_2O_2*	*UVA*	*UVA/20 mmol/L H_2O_2*	*UVA/40 mmol/L H_2O_2*
0	0	0	0	0	0	0	0	0	0
60	5.33	10.16	23.44	1.09	9.69	15.34	1.00	4.37	9.09
120	13.02	19.84	36.50	3.14	17.33	32.19	2.71	10.20	20.22
180	14.20	35.57	49.11	3.96	31.72	43.26	2.90	15.35	29.13

Table S5. *Photodegradation of RY84 (% of removal) after adsorption in wastewater effluent (initial pH 7.5, C_{RY84}=10 mg/L)*

**Time min**	**UVA**	**UVA/40 mmol/L H_2O_2**
0	0	0
60	1.75	13.23
120	7.24	22.71
180	7.65	32.11

REFERENCES

1. Y.-S. Ho, Review of second-order models for adsorption systems. *Journal of hazardous materials* **136**, 681-689 (2006).
2. Y. S. Ho, G. McKay, A Comparison of Chemisorption Kinetic Models Applied to Pollutant Removal on Various Sorbents. *Process Safety and Environmental Protection* **76**, 332-340 (1998).

3. I. Langmuir, The constitution and fundamental properties of solids and liquids. *Journal of American Chemical Society* **38**, 2221-2295 (1916).
4. A. Azari *et al.*, Evaluation of basic violet 16 adsorption from aqueous solution by magnetic zero valent iron-activated carbon nanocomposite using response surface method: Isotherm and kinetic studies. *Journal of Mazandaran University of Medical Sciences* **25**, 333-347 (2015).
5. A. Azari *et al.*, Experimental design, modeling and mechanism of cationic dyes biosorption on to magnetic chitosan-lutaraldehyde composite. *International Journal of Biological Macromolecules* **131**, 633-645 (2019).
6. A. Azari *et al.*, Efficiency of magnitized graphene oxide nanoparticles in removal of 2,4-dichlorophenol from aqueous solution. **26**, 265-281 (2017).
7. M. A. Al-Ghouti, D. A. Da'ana, Guidelines for the use and interpretation of adsorption isotherm models: A review. *Journal of hazardous materials* **393**, 22 (2020).
8. W. Dai, J. Y. Zhang, Y. L. Xiao, W. J. Luo, Z. H. Yang, Dual Function of Modified Palm Leaf Sheath Fibers in Adsorbing Reactive Yellow 3 and Cr(VI) From Dyeing Wastewater. *JOURNAL OF POLYMERS AND THE ENVIRONMENT*.
9. J. R. Nascimento *et al.*, Textile effluent treatment employing yeast biomass and a new nanomagnetic biocomposite. *ENVIRONMENTAL SCIENCE AND POLLUTION RESEARCH* **28**, 27318-27332 (2021).
10. M. Ozacar, I. A. Sengil, Adsorption of reactive dyes on calcined alunite from aqueous solutions. *Journal of Hazardous Materials* **98**, 211-224 (2003).
11. C. De Benedetto, A. Macario, C. Siciliano, J. B. Nagy, P. De Luca, Adsorption of Reactive Blue 116 Dye and Reactive Yellow 81 Dye from Aqueous Solutions by Multi-Walled Carbon Nanotubes. *Materials* **13**, (2020).
12. M. L. Sundar *et al.*, Biochar derived from Caulerpa scalpelliformis for the removal of Reactive Yellow 81 in batch and packed bed column. *Biomass Conversion and Biorefinery*, (2020).
13. M. El Haddad *et al.*, Adsorptive removal of Reactive Yellow 84 dye from aqueous solutions onto animal bone meal. *Journal of Materials and Environmental Science* **3**, 1019-1026 (2012).
14. N. Barka, S. Qourzal, A. Assabbane, A. Nounah, Y. Ait-Ichou, Removal of Reactive Yellow 84 from aqueous solutions by adsorption onto hydroxyapatite. *Journal of Saudi Chemical Society*, 263-267 (2011).
15. H. Abdolmohammad-Zadeh, E. Ghorbani, Z. Talleb, Zinc–aluminum layered double hydroxide as a nano-sorbent for removal of Reactive Yellow 84 dye from textile wastewater effluents. *Journal of the Iranian Chemical Society* **10**, 1103-1112 (2013).
16. M. L. Yola, T. Eren, N. Atar, S. Wang, Adsorptive and photocatalytic removal of reactive dyes by silver nanoparticle-colemanite ore waste. *Chemical Engineering Journal* **242**, 333-340 (2014).
17. U. Filipkowska, T. Jóźwiak, Application of chemically-cross-linked chitosan for the removal of Reactive Black 5 and Reactive Yellow 84 dyes from aqueous solutions. *Journal of Polymer Engineering* **33**, 735-747 (2013).
18. E. D. V. Giordano, M. E. Brassesco, P. Camiscia, G. A. Picó, N. W. Valetti, A New Alternative and Efficient Low-Cost Process for the Removal of Reactive Dyes in Textile Wastewater by Using Soybean Hull as Adsorbent. *Water, Air, & Soil Pollution* **232**, 165 (2021).

19. H. H. Abdel Ghafar, E. K. Radwan, S. T. El-Wakeel, Removal of Hazardous Contaminants from Water by Natural and Zwitterionic Surfactant-modified Clay. *ACS Omega* **5**, 6834-6845 (2020).
20. E. K. Radwan, A. M. Abdel-Aty, S. T. El-Wakeel, H. H. Abdel Ghafar, Bioremediation of potentially toxic metal and reactive dye-contaminated water by pristine and modified Chlorella vulgaris. *Environmental Science and Pollution Research* **27**, 21777-21789 (2020).
21. S. Benkaddour *et al.*, Removal of reactive yellow 145 by adsorption onto treated watermelon seeds: Kinetic and isotherm studies. *Sustainable Chemistry and Pharmacy* **10**, 16-21 (2018).
22. D. Ucar, ADSORPTION OF REMAZOL BLACK RL AND REACTIVE YELLOW 145 FROM AQUEOUS SOLUTIONS BY PINE NEEDLES. *Iranian Journal of Science and Technology-Transactions of Civil Engineering* **38**, 147-155 (2014).
23. E. A. Ofudje *et al.*, Mechanism of Cu(2+)and reactive yellow 145 dye adsorption onto eggshell waste as low-cost adsorbent. *CHEMISTRY AND ECOLOGY* **37**, 268-289 (2021).
24. S. Karmaker, T. Sen, T. K. Saha, Adsorption of reactive yellow 145 onto chitosan in aqueous solution: kinetic modeling and thermodynamic analysis. *Polymer Bulletin* **72**, 1879-1897 (2015).
25. N. Oke, S. Mohan, Development of nanoporous textile sludge based adsorbent for the dye removal from industrial textile effluent. *JOURNAL OF HAZARDOUS MATERIALS* **422**, (2022).
26. A. Lafta *et al.*, Removal of reactive yellow dye 145 from wastewaters over activated carbon that is derived from Iraqi kehdrawy date palm seeds. *World Scientific News* **21**, 124-136 (2015).
27. S. Toutounchi, S. Shariati, K. Mahanpoor, Synthesis of nano-sized magnetite mesoporous carbon for removal of Reactive Yellow dye from aqueous solutions. *APPLIED ORGANOMETALLIC CHEMISTRY* **33**, (2019).
28. H. Younes, H. K. El-Etriby, H. Mahanna, High removal efficiency of reactive yellow 160 dye from textile wastewater using natural and modified glauconite. *INTERNATIONAL JOURNAL OF ENVIRONMENTAL SCIENCE AND TECHNOLOGY*.
29. X. Li *et al.*, Adsorption of reactive yellow X-RG and reactive brilliant red X-3B onto cucurbit[8]uril and cucurbit[6]uril: Effect factors, adsorption behavior and mechanism study. *Journal of Colloid and Interface Science* **498**, 31-46 (2017).
30. A. S. O. Moscofian, C. T. G. V. M. T. Pires, A. P. Vieira, C. Airoldi, Removal of reactive dyes using organofunctionalized mesoporous silicas. *Journal of Porous Materials* **20**, 1179-1188 (2013).
